# Myocardial fat accumulation is associated with cardiac dysfunction in patients with type 2 diabetes, especially in elderly or female patients: a retrospective observational study

**DOI:** 10.1186/s12933-023-01782-y

**Published:** 2023-03-07

**Authors:** Risa Kashiwagi-Takayama, Junji Kozawa, Yoshiya Hosokawa, Sarasa Kato, Satoshi Kawata, Harutoshi Ozawa, Ryohei Mineo, Chisaki Ishibashi, Megu Y. Baden, Ryuya Iwamoto, Kenji Saisho, Yukari Fujita, Sachiko Tamba, Takuya Sugiyama, Hitoshi Nishizawa, Norikazu Maeda, Koji Yamamoto, Masahiro Higashi, Yuya Yamada, Yasushi Sakata, Yuji Matsuzawa, Iichiro Shimomura

**Affiliations:** 1 Department of Metabolic Medicine, Graduate School of Medicine, Osaka University, Suita, Japan; 2 Department of Diabetes Care Medicine, Graduate School of Medicine, Osaka University, Suita, Japan; 3 Department of Lifestyle Medicine, Graduate School of Medicine, Osaka University, Suita, Japan; 4 Department of Endocrinology and Metabolism, Sumitomo Hospital, Osaka, Japan; 5 Department of Metabolism and Atherosclerosis, Graduate School of Medicine, Osaka University, Suita, Japan; 6 Department of Radiology, National Hospital Organization Osaka National Hospital, Osaka, Japan; 7 Cardiovascular Medicine, Graduate School of Medicine, Osaka University, Suita, Japan

**Keywords:** Ectopic fat, Type 2 diabetes, Myocardium, Myocardial function, Visceral obesity

## Abstract

**Background:**

Ectopic fat is fat that accumulates in or around specific organs or compartments of the body including myocardium. The clinical features of type 2 diabetes patients with high fat accumulation in the myocardium remain unknown. Moreover, little is known about the influence of myocardial fat accumulation in type 2 diabetes on coronary artery disease and cardiac dysfunction. We aimed to clarify the clinical features, including cardiac functions, of type 2 diabetes patients with myocardial fat accumulation.

**Methods:**

We retrospectively enrolled type 2 diabetes patients who underwent ECG-gated coronary computed tomography angiography (CCTA) and abdominal computed tomography (CT) scan examinations within 1 year of CCTA from January 2000 to March 2021. High fat accumulation in the myocardium was defined as the low mean myocardial CT value of three regions of interest, and the associations between CT values and clinical characteristics or cardiac functions were assessed.

**Results:**

In total, 124 patients were enrolled (72 males and 52 females). The mean age was 66.6 years, the mean BMI was 26.2 kg/m^2^, the mean ejection fraction (EF) was 67.6%, and the mean myocardial CT value was 47.7 Hounsfield unit. A significant positive correlation was found between myocardial CT value and EF (r = 0.3644, p = 0.0004). The multiple regression analyses also showed that myocardial CT value was independently associated with EF (estimate, 0.304; 95% confidence interval (CI) 0.092 to 0.517; p = 0.0056). Myocardial CT value showed significant negative correlations with BMI, visceral fat area and subcutaneous fat area (r = − 0.1923, − 0.2654, and -0.3569, respectively, p < 0.05). In patients who were ≥ 65 years or female, myocardial CT value showed significant positive correlations with not only EF (r = 0.3542 and 0.4085, respectively, p < 0.01) but also early lateral annular tissue Doppler velocity (Lat e’) (r = 0.5148 and 0.5361, respectively, p < 0.05). The multiple regression analyses showed that myocardial CT value was independently associated with EF and Lat e’ in these subgroups (p < 0.05).

**Conclusions:**

Patients with type 2 diabetes, especially in elderly or female patients, who had more myocardial fat had more severe left ventricular systolic and diastolic dysfunctions. Reducing myocardial fat accumulation may be a therapeutic target for type 2 diabetes patients.

**Supplementary Information:**

The online version contains supplementary material available at 10.1186/s12933-023-01782-y.

## Introduction

Ectopic fat is fat that accumulates in or around specific organs or compartments of the body [1]. According to recent studies, fat accumulations are observed in organs such as liver, skeletal muscle, kidney, heart, and pancreas [2–5]. In the heart, epicardial fat is another type of ectopic fat. Epicardial fat is increased in patients with type 2 diabetes [6], and its accumulation is associated with the presence of coronary artery disease and cardiac arrhythmias [7–9]. Moreover, the accumulation of triglycerides (TGs) in coronary arteries has been recently reported [10, 11]. Ectopic fat is also observed in the myocardium. Myocardial fat accumulation has been assessed using proton magnetic resonance (MR) spectroscopy, and myocardial TG content in obesity, metabolic syndrome or type 2 diabetes patients was increased compared with that in control subjects [12, 13]. Myocardial fat accumulation has been reported to be associated with higher left ventricular mass in healthy subjects [14] as well as in heart failure patients [15], and, among Blacks with diabetes, high left ventricular mass is associated with worse outcomes, indicating subclinical myocardial dysfunction [16]. However, the clinical features of type 2 diabetes patients with high fat accumulation in the myocardium remain unknown. Moreover, little is known about the influence of myocardial fat accumulation in type 2 diabetes on coronary artery disease and myocardial systolic or diastolic dysfunction.

Not only MR spectroscopy but also noncontrasted computed tomography (CT) attenuation values (Hounsfield units) are used to assess the fat accumulation in the liver, pancreas, and skeletal muscle [17, 18]. It has been reported that CT values of the myocardium were apparently low in two patients with myocardial TG deposition compared to control subjects [19], indicating that this modality could be applicable to the myocardium. The present study was designed to clarify the clinical features of type 2 diabetes patients with myocardial fat accumulation assessed by CT values. This study also investigated the associations between myocardial fat accumulation and coronary artery disease or myocardial function in type 2 diabetes.

## Methods

### Patients

We searched the database of patients with type 2 diabetes who were referred for ECG-gated coronary computed tomography angiography (CCTA) examinations (Toshiba Aquilion CT scanner, Toshiba Medical, Tochigi, Japan; SOMATOM Definition or Force, Siemens Healthineers, Forchheim, Germany) for the first time and underwent abdominal CT scans (Toshiba Aquilion CT scanner, Toshiba Medical, Tochigi, Japan; Discovery CT750 HD, General Electric Healthcare, Chicago, IL, USA; SOMATOM Definition, Siemens Healthineers, Forchheim, Germany; GE Optima CT660, General Electric Healthcare, Chicago, IL, USA) within 1 year of CCTA at Osaka University Hospital or Sumitomo Hospital between January 2000 and March 2021. A total of 411 patients met these criteria. Among these patients, we excluded patients to avoid the influence of cardiac function or the myocardial CT value of pathological conditions other than myocardial fat accumulation. The excluded patients included those with heart failure with reduced ejection fraction (≤ 40%), those with valvular heart disease and those who had received past percutaneous coronary intervention (PCI) for coronary artery disease. Moreover, we excluded patients who had liver cirrhosis, renal failure (estimated glomerular filtration rate of < 30 mL/min/1.73 m^2^), malignant diseases and diseases requiring glucocorticoids for the treatment of other diseases. Furthermore, it is known that changes in X-ray tube voltage affect CT attenuation values [20]. Therefore, patients who underwent CCTA or abdominal CT examinations that were not performed at 120 kV were excluded. Using these criteria, 124 patients were finally included in our analyses. The flowchart for the recruitment of the patients is shown in Additional file 1: Fig S1. Among these 124 patients, the medications for diabetes at the time of CCTA were as follows: insulin for 42 patients, glucagon-like peptide-1 (GLP-1) receptor agonists for 9 patients, sulfonylureas for 31 patients, biguanides for 43 patients, dipeptidyl peptidase-4 inhibitors for 40 patients, α-glucosidase inhibitors for 26 patients, thiazolidinediones for 13 patients, glinides for 12 patients and sodium–glucose cotransporter 2 (SGLT2) inhibitors for 3 patients. The medications for dyslipidemia at the time of CCTA were as follows: statins for 69 patients, fibrates for 9 patients, ezetimibe for 5 patients, omega-3 fatty acids for 8 patients and probucol for 1 patient.

This study was approved by the Institutional Ethics Review Boards of Osaka University Hospital and Sumitomo Hospital and was carried out in accordance with the principles of the Declaration of Helsinki. The study was announced to the public on the websites of our department at Osaka University Hospital and Sumitomo Hospital, and all patients were allowed to participate or refuse to participate in the study.

### Clinical parameters

We obtained the following data at the time of the first CCTA examinations from medical records: age, sex, body mass index (BMI), previous highest BMI, waist circumference, systolic and diastolic blood pressure, levels of hemoglobin A1c (HbA1c), fasting plasma glucose (FPG), C-peptide index (CPI), homeostasis model assessment of β-cell function (HOMA-β), homeostasis model assessment of insulin resistance (HOMA-IR), total cholesterol (T-Chol), triglycerides (TGs), high-density lipoprotein cholesterol (HDL-C), low-density lipoprotein cholesterol (LDL-C), uric acid (UA), aspartate transaminase (AST), alanine transaminase (ALT), γ-glutamyltranspeptidase (γGTP), estimated glomerular filtration rate (eGFR), brain natriuretic peptide (BNP), and serum total adiponectin levels. CPI was defined as F-CPR (nmol/L) × 100/FPG (mmol/L), HOMA-β as F-IRI (µIU/mL) × 20/(FPG [mmol/L]—3.5), HOMA-IR as F-IRI (μU/mL) × FPG (mg/dL)/405. Serum total adiponectin levels were measured by the latex particle-enhanced turbidimetric immunoassay (human adiponectin latex kit; Otsuka Pharmaceutical Co., Ltd., Tokyo, Japan).

We also obtained the following echocardiographic data at the time of the first CCTA examinations from medical records: ejection fraction (EF), maximum inferior vena cava diameters (IVC_max_), early lateral annular tissue Doppler velocity (Lat e’), septum mitral early diastolic velocity/early lateral annular tissue Doppler velocity (sep E/e’), mean mitral early diastolic velocity/early lateral annular tissue Doppler velocity (mean E/e’), and wall motion abnormalities. Echocardiographic studies were performed with commercially available equipment.

CCTA images were visually interpreted by radiologists. We obtained the data of the presence of stenosis of ≥ 50% in at least one coronary arterial segment. Finally, we obtained the clinical history of percutaneous coronary intervention (PCI) or coronary artery bypass graft (CABG) surgery after the first CCTA examinations until April 2021.

### Measurement of myocardial, pancreatic, liver and iliopsoas muscle fat

It has been shown that unenhanced CT values are well correlated with the degree of fat content in the pancreas, liver and iliopsoas muscle [17, 21, 22]. The myocardial CT value (H) was defined as the mean CT value of three regions of interest with areas of 10 mm^2^ in two different parts of the left ventricular free wall and one part of the myocardial septum. To confirm the intramyocardial measurement areas excluding blood pool and epicardial fat, we compared contrasted and noncontrasted images side-by-side, as shown in Additional file 1: Figure S2. High fat accumulation in the myocardium was defined as a low CT value (low H). The first reader performed CT measurements of all cases. To confirm the inter- and intra-observer variabilities, the second investigator who was blinded to clinical data analyzed CT values of all cases independently. Inter-observer and intra-observer variabilities were examined with the intraclass correlation coefficient (ICC) (2,1) and (1,1), respectively. The measurement of the myocardial CT value showed good inter- and intra-observer variabilities {ICC (2,1) = 0.92 and ICC (1,1) = 0.90}.

The pancreatic CT value (P) was defined as the mean CT value of three regions of interest with areas of 1 cm^2^ in three different pancreatic parts, the head, body and tail, as previously shown [25]. We also defined a liver CT value (L) as the mean CT value of three regions of interest with areas of 1 cm^2^ in three different segments of liver: anterior, posterior and lateral [25]. The CT value of the iliopsoas muscle (M) was defined as the mean CT value of the regions of interest with areas of 1 cm^2^ in iliopsoas muscles on both the left and right sides at the umbilical level. Similar methods were previously used in the study of paraspinal muscle density [23, 24]. We defined a splenic CT value (S) as the mean of three regions of interest with areas of 1 cm^2^ in three different splenic levels: upper, middle and lower [25]. Both pancreatic and hepatic attenuation measurements were analyzed with normalization with the spleen [17, 25, 26]. As previously shown, indices of pancreatic and hepatic fat content were defined as the differences between the pancreatic and splenic CT values (P-S) and the liver and splenic CT values (L-S), respectively [25]. High fat accumulations in the pancreas and liver were defined as low CT values (low P-S and low L-S, respectively). The first reader performed CT measurements of all cases, and the second investigator who was blinded to clinical data analyzed CT values of L-S, P-S, M and S of all cases independently. The measurements of L-S, P-S, M and S showed good inter- and intra-observer variabilities {L-S, ICC (2,1) = 0.94 and ICC (1,1) = 0.98; P-S, ICC (2,1) = 0.87 and ICC (1,1) = 0.97; M, ICC (2,1) = 0.94 and ICC (1,1) = 0.96; S, ICC (2,1) = 0.95 and ICC (1,1) = 0.96}.

The images were analyzed using the software program Synapse viewer (Fujifilm Inc., Tokyo, Japan). The visceral fat area (VFA) and subcutaneous fat area (SFA) were computed or measured manually using commercial software for CT scans taken at the umbilical level in a supine position, based on Japanese guidelines for obesity treatment (Japan Society for the Study of Obesity, in Japanese) [27].

### Statistical analysis

Data are presented as the mean ± standard deviation or number of patients and compared by the Student’s t-test or Pearson’s chi-square test. The relationships between the myocardial CT value and clinical parameters or CT values of each organ were assessed using Pearson’s correlation coefficient analyses. The factors that contributed to EF or Lat e’ were assessed using multiple regression analyses. We did not impute the missing data, and we performed complete case analyses. The association between coronary arteries with ≥ 50% stenosis in CCTA images and the myocardial CT value was evaluated using multiple logistic regression analyses. We also performed multiple logistic regression analyses to evaluate the relationship between the clinical history of PCI or CABG surgery after the first CCTA examinations until April 2021 and the myocardial CT value. All statistical analyses were performed using JMP Pro 14 software (SAS Institute Inc., Cary, NC, USA). P values < 0.05 were considered statistically significant.

## Results

### Correlation analyses between clinical parameters and myocardial fat content

**Fig. 1:**
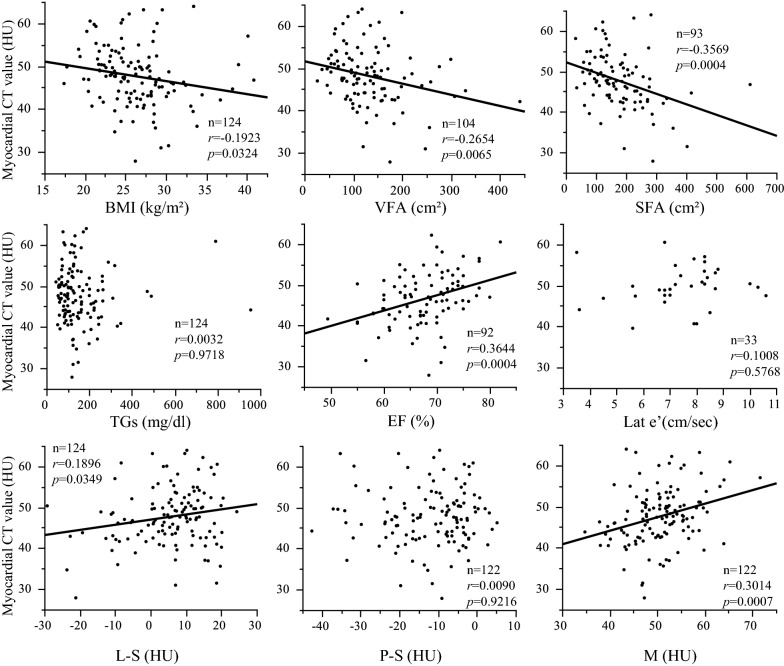
Correlation analysis of myocardial CT values (an index of myocardial fat content) and clinical parameters. The myocardial CT value was associated with EF. Furthermore, the myocardial CT value was associated with BMI, VFA, SFA, L-S and M. *BMI* body mass index, *VFA* visceral fat area, *SFA* subcutaneous fat area, *TGs*, triglycerides, *EF* ejection fraction, *Lat e’* early lateral annular tissue Doppler velocity, *S* splenic CT value, *L* liver CT value, *P* pancreatic CT value, *M* iliopsoas muscle CT value

Table 1 summarizes the baseline clinical characteristics of the patients at the first CCTA examination. The results of the correlation analyses between myocardial fat content represented by H and clinical parameters are shown in Fig. 1. In analyses of all patients, H showed significant negative correlations with BMI, VFA and SFA. In other words, patients with higher levels of obesity had more myocardial fat. With regard to the echocardiographic data, a significant positive correlation was found between H and EF. That is, patients with more myocardial fat had more severe left ventricular systolic dysfunction. There was no significant correlation between H and echocardiographic parameters of diastolic dysfunction (Lat e’). H also showed a significant positive correlation with L-S and M. No significant correlation was found between H and P-S. The multiple regression analyses showed that H was independently associated with EF (estimate, 0.304; 95% confidence interval (CI) 0.092 to 0.517; p < 0.05) (Table 2). There was no significant correlation between H and glycemic parameters. No significant correlation was found between H and TGs, and the result was consistent after a log transformation of TGs.

**Table 1:** Clinical characteristics of patients

		N
Age (years)	66.6 ± 10.1	124
Sex (male/female)	72/52	124
BMI (kg/m^2^)	26.2 ± 4.4	124
Waist circumference (cm)	94.8 ± 10.3	61
Previous highest BMI (kg/m^2^)	29.4 ± 4.4	91
Systolic blood pressure (mmHg)	141 ± 21	111
Diastolic blood pressure (mmHg)	79 ± 15	111
FPG (mg/dl)	148 ± 45	120
HOMA-β	42.3 ± 45.3	69
CPR index	1.28 ± 0.95	83
HOMA-IR	2.6 ± 1.7	69
HbA1c (%; mmol/mol)	8.0 ± 1.8; 67 ± 19.7	124
Adiponectin (μg/ml)	8.5 ± 5.6	36
T-chol (mg/dl)	199 ± 36	121
HDL-chol (mg/dl)	55 ± 17	124
LDL-chol (mg/dl)	118 ± 32	120
TGs (mg/dl)	153 ± 122	124
UA (mg/dl)	5.5 ± 1.3	123
AST (U/L)	27 ± 19	124
ALT (U/L)	27 ± 20	124
γGTP (U/L)	53 ± 70	123
eGFR (ml/min/1.73 m^2^)	73.3 ± 19.4	120
BNP (pg/ml)	35.8 ± 42.5	69
EF (%)	67.6 ± 6.2	92
IVC_max_	11.9 ± 3.4	86
Lat e’ (cm/sec)	7.4 ± 1.7	33
Sep E/e’	12.0 ± 4.5	57
Mean E/e’	11.0 ± 3.7	36
Wall motion abnormalities (Yes/No)	7/85	92
Myocardial CT value (HU)	47.7 ± 6.9	124
Liver CT value (HU)	53.2 ± 10.7	124
Liver CT value minus splenic CT value (HU)	5.2 ± 10.4	124
Pancreatic CT value (HU)	35.8 ± 9.4	122
Pancreatic CT value minus splenic CT value (HU)	− 12.2 ± 10.2	122
Iliopsoas muscle CT value (HU)	50.5 ± 6.3	122
Splenic CT value (HU)	47.9 ± 4.7	124
VFA (cm^2^)	131.9 ± 68.0	104
SFA (cm^2^)	176.0 ± 92.3	93
Coronary arteries with ≥ 50% stenosis in CCTA images (Yes/No)	75/49	124
Clinical history of PCI or CABG surgery after the first CCTA (Yes/No)	41/83	124

Data are presented as the mean ± standard deviation or number of participants (N)

*BMI* body mass index, *HbA1c* the levels of hemoglobin A1c, *FPG* fasting plasma glucose, *CPI* C-peptide index, *HOMA-β* homeostasis model assessment of β-cell function, *HOMA-IR* homeostasis model assessment of insulin resistance, *T-Chol* total cholesterol, *TGs* triglycerides, *HDL-C* high-density lipoprotein cholesterol, *LDL-C* low-density lipoprotein cholesterol, *UA* uric acid, *AST* aspartate transaminase, *ALT* alanine transaminase, *γGTP* γ-glutamyltranspeptidase, *eGFR* estimated glomerular filtration rate, *BNP* brain natriuretic peptide, *EF* ejection fraction, *IVC*_*max*_ maximum inferior vena cava diameters, *Lat e’* early lateral annular tissue Doppler velocity, *Sep E/e’* septum mitral early diastolic velocity/early lateral annular tissue Doppler velocity, *Mean E/e’* mean mitral early diastolic velocity/early lateral annular tissue Doppler velocity, *VFA* visceral fat area, *SFA* subcutaneous fat area, *CCTA* coronary computed tomography angiography

**Table 2:** Multiple regression analysis (total)

Variables	Estimate	Standard Error	t-value	P-value	95% Confidence Interval	
					Lower	Upper
Multiple regression analysis for ejection fraction (EF) of subjects with complete data (N = 78)						
Myocardial CT value	0.304	0.107	2.85	0.0056	0.092	0.517
VFA	− 0.005	0.010	− 0.49	0.6242	− 0.025	0.015
Liver CT value minus splenic CT value	− 0.041	0.076	− 0.54	0.5934	− 0.193	0.111

N, the number of patients; VFA, visceral fat area

### Correlation analyses between clinical parameters and myocardial fat content based on age

**Fig. 2:**
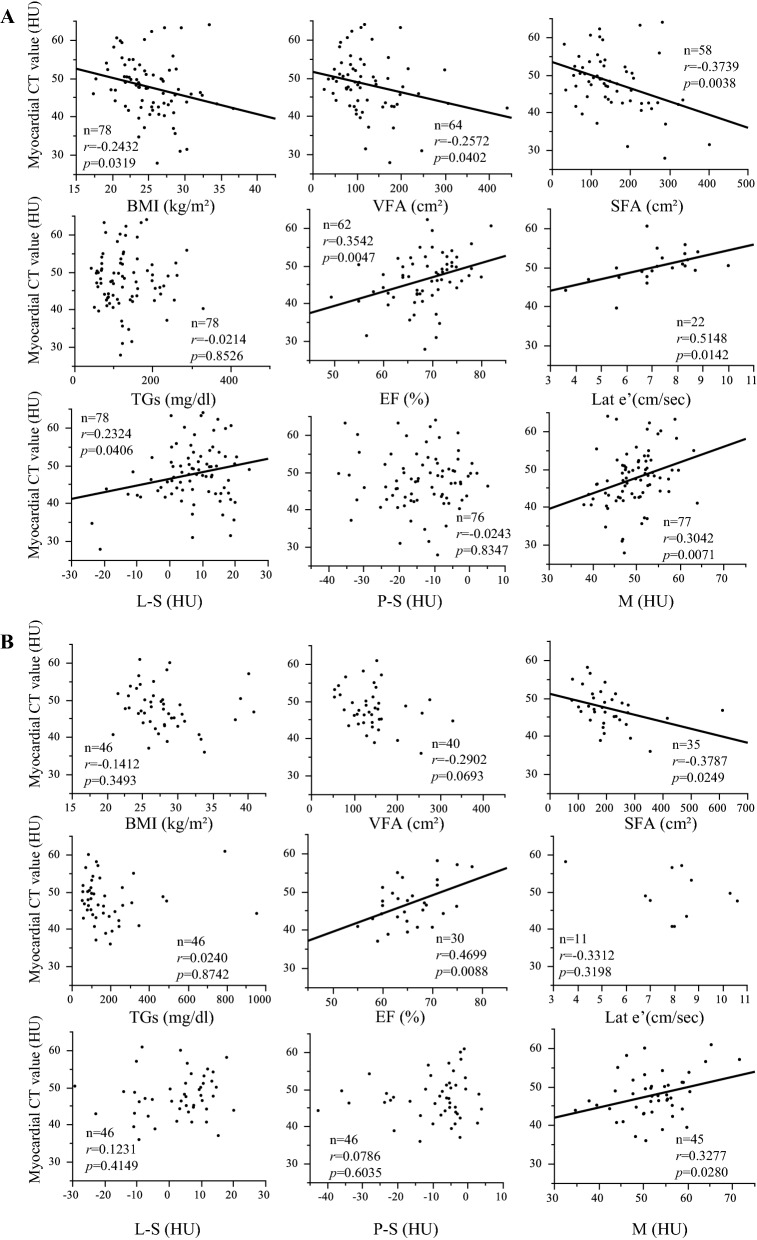
Correlation analysis between myocardial CT values (an index of myocardial fat content) and clinical parameters of the ≥ 65 years (the older subgroup) (**A**) and < 65 years (the younger subgroup) (**B**) subgroups. In the older subgroup, the myocardial CT value was associated with EF and Lat e’. Furthermore, the myocardial CT value was associated with BMI, VFA, SFA, L-S and M. In the younger subgroup, the myocardial CT value was associated with EF. Furthermore, the myocardial CT value was associated with SFA and M. *BMI* body mass index, *VFA* visceral fat areas, *SFA* subcutaneous fat areas, *TGs* triglycerides, *EF* ejection fraction, *Lat e’* early lateral annular tissue Doppler velocity, *S* splenic CT value, *L* liver CT value, *P* pancreatic CT value, *M* iliopsoas muscle CT value

A previous study reported that there are age-related changes in the echocardiographic parameters of diastolic function [28]. Therefore, we divided the patients into two subgroups: the ≥ 65 years (the older subgroup) and < 65 years (the younger subgroup) subgroups. The clinical characteristics of each subgroup are shown in Additional file 1: Table S1. Figure 2 shows the results of the correlation analyses between myocardial fat content represented by H and clinical parameters of the older (≥ 65 years) and younger (< 65 years) subgroups. H showed significant negative correlations with BMI, VFA and SFA in the older subgroup. H showed a significant negative correlation with SFA and had a tendency of negative correlation with VFA in the younger subgroup. With regard to the echocardiographic data, significant positive correlations were found between H and EF in both subgroups. In addition, in the older subgroup, there was a significant positive correlation between H and Lat e', and the multiple regression analyses showed that H was independently associated with EF and Lat e’ (estimate, 0.312 and 0.184, respectively; 95% CI 0.058 to 0.565 and 0.036 to 0.332, respectively; p < 0.05) (Table 3). In other words, the older subgroup with more myocardial fat had more severe left ventricular systolic and diastolic dysfunctions. In the younger subgroup, there was no significant correlation between H and Lat e’, and the multiple regression analysis showed that H was independently associated with EF (estimate, 0.421; 95% CI 0.003 to 0.838; p < 0.05) (Table 3). H also showed a significant positive correlation with L-S and M in the older subgroup, and there was a significant correlation between H and M in the younger subgroup. No significant correlation was found between H and P-S in either subgroup. There was no significant correlation between H and glycemic parameters in either subgroup. No significant correlation was found between H and TGs, and the result was consistent after a log transformation of TGs in either subgroup.

**Table 3:** Multiple regression analyses (older, younger)

Variables	Estimate	Standard Error	t-value	P-value	95% Confidence Interval	
					Lower	Upper
Multiple regression analysis for ejection fraction (EF) in the older subgroup with complete data (N = 52)						
Myocardial CT value	0.312	0.126	2.47	0.0171	0.058	0.565
VFA	− 0.004	0.012	− 0.39	0.6965	− 0.029	0.020
Liver CT value minus splenic CT value	− 0.048	0.101	− 0.47	0.6372	− 0.250	0.154
Multiple regression analysis for early lateral annular tissue Doppler velocity (Lat e') in the older subgroup with complete data (N = 22)						
Myocardial CT value	0.184	0.071	2.61	0.0178	0.036	0.332
VFA	0.005	0.007	0.67	0.5134	− 0.010	0.020
Liver CT value minus splenic CT value	− 0.027	0.046	− 0.59	0.5628	− 0.125	0.070
Multiple regression analysis for ejection fraction (EF) in the younger subgroup with complete data (N = 26)						
Myocardial CT value	0.421	0.201	1.09	0.0483	0.003	0.838
VFA	− 0.007	0.019	− 0.35	0.7275	− 0.047	0.033
Liver CT value minus splenic CT value	− 0.159	0.120	− 1.33	0.1979	− 0.408	0.090

*N* the number of patients, *VFA* visceral fat area

### Correlation analyses between clinical parameters and myocardial fat content based on sex

**Fig. 3:**
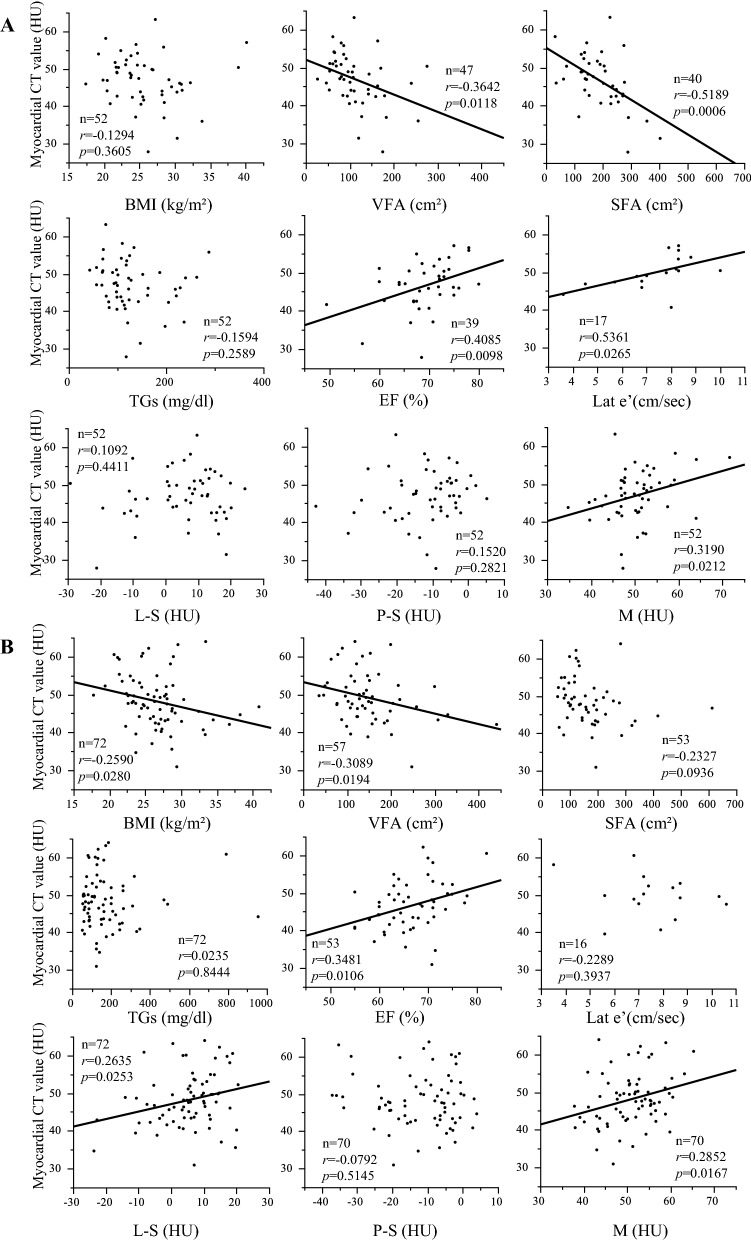
Correlation analysis between myocardial CT values (an index of myocardial fat content) and clinical parameters of the female subgroup (**A**) and the male subgroup (**B**). In the female subgroup, the myocardial CT value was associated with EF and Lat e’. Furthermore, the myocardial CT value was associated with VFA, SFA and M. In the male subgroup, the myocardial CT value was associated with EF. Furthermore, the myocardial CT value was associated with BMI, VFA, L-S and M. *BMI* body mass index, *VFA* visceral fat area, *SFA* subcutaneous fat area, *TGs* triglycerides, *EF* ejection fraction, *Lat e’* early lateral annular tissue Doppler velocity, *S* splenic CT value, *L* liver CT value, *P* pancreatic CT value, *M* iliopsoas muscle CT value

We divided patients into sex (female and male) subgroups and analyzed the correlation between H and clinical parameters. The clinical characteristics of each subgroup are shown in Additional file 1: Table S2. The results of the correlation analyses between the myocardial fat content represented by H and clinical parameters of the female and male subgroups are shown in Fig. 3. In the female subgroup, H showed significant negative correlations with VFA and SFA, while H showed significant negative correlations with BMI and VFA in the male subgroup. With regard to the echocardiographic data, a significant positive correlation was found between H and EF in both subgroups. In addition, in the female subgroup, there was a significant positive correlation between H and Lat e’, and the result of the significant association of myocardial fat and Lat e’ in female subgroup was consistent after we excluded two women of premenopausal age (40 and 48 years) (r = 0.5451, p = 0.0290). The multiple regression analyses showed that H was independently associated with EF and Lat e’ (estimate, 0.346 and 0.196, respectively; 95% CI 0.070 to 0.622 and 0.013 to 0.379, respectively; p < 0.05) (Table 4). That is, the female subgroup with more myocardial fat had more severe left ventricular systolic and diastolic dysfunction. In the male subgroup, there was no significant association between H and Lat e’, and the multiple regression analysis showed that H was not independently associated with EF (Table 4). H also showed significant positive correlations with M in the female subgroup and with L-S and M in the male subgroup. Furthermore, no significant correlation was found between H and P-S in either subgroup. There was no significant correlation between H and glycemic parameters in either subgroup. No significant correlation was found between H and TGs, and the result was consistent after a log transformation of TGs in either subgroup.

**Table 4:** Multiple regression analyses (female, male)

Variables	Estimate	Standard Error	t-value	P-value	95% Confidence Interval	
					Lower	Upper
Multiple regression analysis for ejection fraction (EF) in the female subgroup with complete data (N = 36)						
Myocardial CT value	0.346	0.134	2.56	0.0156	0.070	0.622
VFA	− 0.015	0.019	− 0.75	0.4585	− 0.054	0.025
Liver CT value minus splenic CT value	− 0.173	0.084	− 2.05	0.0488	− 0.345	− 0.001
Multiple regression analysis for early lateral annular tissue Doppler velocity (Lat e') in the female subgroup with complete data (N = 17)						
Myocardial CT value	0.196	0.085	2.31	0.0377	0.013	0.379
VFA	0.009	0.011	0.78	0.4487	− 0.015	0.032
Liver CT value minus splenic CT value	0.027	0.047	0.56	0.5847	− 0.076	0.129
Multiple regression analyses for EF in the male subgroup with complete data (N = 42)						
Myocardial CT value	0.267	0.167	1.60	0.1180	− 0.071	0.605
VFA	0.012	0.012	1.04	0.3070	− 0.012	0.038
Liver CT value minus splenic CT value	0.164	0.132	1.24	0.2214	− 0.103	0.432

*N* the number of patients, *VFA* visceral fat area

### Association between coronary atherosclerosis or clinical history of PCI or CABG surgery and myocardial fat content

The multiple logistic regression analyses of the relationship between the myocardial fat content represented by H and coronary arteries with ≥ 50% stenosis in CCTA images showed no significant correlation (Additional file 1: Fig S3). Likewise, there was no significant association between H and clinical history of PCI or CABG surgery after the first CCTA examinations (Additional file 1: Fig S3).

## Discussion

The present study showed that myocardial fat in type 2 diabetes patients was strongly associated with left ventricular systolic dysfunction. Furthermore, this study demonstrated that myocardial fat in type 2 diabetes patients who were ≥ 65 years or female had a strong association with left ventricular diastolic dysfunction, evaluated by Lat e’ as was previously reported [29]. In addition, we showed that myocardial fat in type 2 diabetes patients was strongly associated with obesity and hepatic and iliopsoas muscle fat.

### Relationships between myocardial fat and left ventricular dysfunctions

To our knowledge, this is the first study that demonstrated that myocardial fat of type 2 diabetes patients had strong associations with not only left ventricular diastolic dysfunction but also left ventricular systolic dysfunction. It has been reported that the accumulation of myocardial fat is associated with left ventricular diastolic dysfunction by using ^1^H- MR spectroscopy; however, no association was found between myocardial fat accumulation and left ventricular ejection fraction [30]. This inconsistency might be derived from the relatively large number of enrolled patients restricted to type 2 diabetes patients in our study, while the relatively small number of patients, including not only type 2 diabetes patients but also healthy volunteers, were included in that study [30].

Mitochondrial dysfunction in type 2 diabetes mellitus has been observed throughout various organ systems [31]. Sixty to ninety percent of cardiac energy is generated by the β-oxidation of fatty acids [32]. Therefore, myocardial mitochondrial dysfunction may lead to the accumulation of myocardial fat. Moreover, the adipose triglyceride lipase (ATGL) activity in the peripheral leucocytes of type 2 diabetes patients has been reported to be lower than that of the healthy group [33]. If ATGL activity in the cardiomyocytes of type 2 diabetes patients is also reduced, this may be related to the accumulation of myocardial fat. These possible mechanisms may lead to a decrease in energy production and an increase in fat accumulation in the myocardium, resulting in left ventricular systolic dysfunction in type 2 diabetes patients. In this study, no significant relationship was found between the use of SGLT2 inhibitors or GLP-1 receptor agonists and myocardial CT value (data not shown), though these medications have been reported to improve cardiac functions in human diabetic hearts [34] and in a mouse model of heart failure with preserved ejection fraction [35]. Because this study is cross-sectional and the limited number of the patients with these medications were included, further studies are needed to investigate the influence of these medications on myocardial fat accumulation.

We also found that myocardial fat in type 2 diabetes patients who were ≥ 65 years or female had a significant association with left ventricular diastolic dysfunction represented by the decrease in Lat e’. Heart failure with preserved ejection fraction (HFpEF) is more prevalent in elderly individuals and women [36, 37], but there were no statistically significant differences in Lat e’ in the age and sex subgroups compared by analysis of variance (p = 0.2003 and 0.9921, respectively). The higher prevalence of HFpEF in elderly patients implies the effects of aging on myocardial structure (the high prevalence of cardiac hypertrophy), endothelial inflammation and vascular inflammation [38, 39]. In addition, the importance of 17β-estradiol (E2) is considered to be critical in the onset of HFpEF in women after menopause [36]. Although the mechanism remains unclear, the present study might imply the contribution of myocardial fat to left ventricular diastolic dysfunction in elderly and female patients with type 2 diabetes.

### Relationships between myocardial fat and obesity related factors

Moreover, we showed that myocardial fat was strongly associated with abdominal obesity and hepatic fat in patients with type 2 diabetes. Obesity is associated with pericardial or epicardial adipose tissue, which is associated with subclinical left ventricular functional deterioration or HFpEF [40, 41]. In this study, the younger subgroup had higher BMI than the older subgroup, which might have led to higher insulin resistance evaluated by HOMA-IR. The results might be reflected by higher triglyceride level as well as elevated level of liver enzymes and lower liver CT value (Additional file 1: Table S1). Cardiac insulin resistance is considered to have a predominant role in determining alterations of left ventricular mechano-energetic performance [42]. However, no significant correlation was found between HOMA-IR and myocardial CT value or cardiac function in the younger subgroup. Further studies are needed to investigate the influence of insulin resistance on myocardial fat accumulation as well as cardiac function.

In addition, another study reported that obesity- or metabolic syndrome-related alterations in lipid metabolism increase myocardial fat content, epicardial fat thickness, inflammation and oxidative stress which ultimately leads to cardiac lipotoxicity and diastolic dysfunction [43]. Lipotoxicity derived from myocardial lipid accumulation may partially contribute to cardiac dysfunction [44], as shown in this study. Moreover, it has been reported that targeting cholesteryl ester accumulation in the heart improves cardiac insulin response [45]. Thus, fat accumulation including TGs as well as cholesteryl ester accumulation in myocardium may be a therapeutic target for type 2 diabetes patients with heart failure.

### Relationship between myocardial fat and coronary atherosclerosis

The current study showed no significant association between myocardial fat accumulation and coronary arteries with ≥ 50% stenosis in CCTA images. Moreover, no significant association was found between myocardial fat and clinical history of PCI or CABG surgery after the first CCTA examinations. It has recently been proposed that coronary microvascular dysfunction participates in HFpEF development [46]. We only evaluated coronary macrovascular lesions by CCTA images and the history of PCI or CABG surgery, and therefore, we cannot deny the possibility that myocardial fat accumulation has some impact on microvascular dysfunction. Further studies are needed to investigate the influence of myocardial fat on coronary atherosclerosis.

## Limitations

First, the sample size of the study was small, especially when we divided patients into age or sex subgroups. Second, the inclusion criteria of type 2 diabetes patients who underwent CCTA may imply selection bias toward patients with suspected heart diseases. Therefore, the results of our study may not be applicable to all patients with type 2 diabetes. Third, only Lat e’ was used to evaluate left ventricular diastolic function, and other echocardiographic data were not used in this study. However, we do think that our method using Lat e’ is appropriate to investigate the relationship between myocardial fat content and left ventricular diastolic function because the other parameters, such as the E/A ratio, do not change in a linear manner according to the severity of diastolic dysfunction [47]. Finally, we could not evaluate the effect of hypertension or dyslipidemia and the medications for them on the cardiac parameters in the analyses.

## Conclusions

Our study is the first to show that myocardial fat in type 2 diabetes patients has a strong association with left ventricular systolic dysfunction. Moreover, myocardial fat is associated with left ventricular diastolic dysfunction in ≥ 65-year-old or female patients with type 2 diabetes. Reducing myocardial fat accumulation may be a therapeutic target for type 2 diabetes patients with heart failure.

## Supplementary Information

**Additional file 1**: **Table S1**. Clinical characteristics of older and younger subgroups. **Table S2**. Clinical characteristics of female and male subgroups. **Table S3**. Clinical characteristics of patients with complete cases for multiple regression analysis (total). **Table S4**. Clinical characteristics of older and younger subgroups with complete cases for multiple regression analysis. **Table S5**. Clinical characteristics of female and male subgroups with complete cases for multiple regression analysis. **Figure S1** Flowchart for the recruitment of the patients. **Figure S2**. Method for measuring myocardial CT values on non-contrasted CT images. **Figure S3**. Multiple logistic regression analyses (total).

## Data Availability

All data relevant to the study are included in the article. The datasets generated and analyzed during the current study are available from the corresponding author upon reasonable request.

## Abbreviations

BMI: Body mass index
WC: Waist circumference
HbA1c: The levels of hemoglobin A1c
FPG: Fasting plasma glucose
CPI: C-peptide index
HOMA-β: Homeostasis model assessment of β-cell function
HOMA-IR: Homeostasis model assessment of insulin resistance
T-Chol: Total cholesterol
TGs: Triglycerides
HDL-C: High-density lipoprotein cholesterol
LDL-C: Low-density lipoprotein cholesterol
UA: Uric acid
AST: Aspartate transaminase
ALT: Alanine transaminase
γGTP: γ-Glutamyltranspeptidase
eGFR: Estimated glomerular filtration rate
BNP: Brain natriuretic peptide
EF: Ejection fraction
IVC_max_: Maximum inferior vena cava diameters
Lat e’: Early lateral annular tissue Doppler velocity
Sep E/e’: Septum mitral early diastolic velocity/early lateral annular tissue Doppler velocity
Mean E/e’: Mean mitral early diastolic velocity/early lateral annular tissue Doppler velocity
H: Myocardial CT value
S: Splenic CT value
L: Liver CT value
P: Pancreatic CT value
M: Iliopsoas muscle CT value
VFA: Visceral fat area
SFA: Subcutaneous fat area
CCTA: Coronary computed tomography angiography
