# Supplementary material for: Myocardial fat accumulation is associated with cardiac dysfunction in patients with type 2 diabetes, especially in elderly or female patients: a retrospective observational study
Source: Cardiovasc Diabetol. 2023 Mar 7;22:48. doi: 10.1186/s12933-023-01782-y (PMC9993532; doi:10.1186/s12933-023-01782-y)
Supplement: Supplementary file 1 — Additional file 1: Table S1. Clinical characteristics of older and younger subgroups. Table S2. Clinical characteristics of female and male subgroups. Table S3. Clinical characteristics of patients with complete cases for multiple regression analysis (total). Table S4. Clinical characteristics of older and younger subgroups with complete cases for multiple regression analysis. Table S5. Clinical characteristics of female and male subgroups with complete cases for multiple regression analysis. Figure S1 Flowchart for the recruitment of the patients. Figure S2. Method for measuring myocardial CT values on non-contrasted CT images. Figure S3. Multiple logistic regression analyses (total). [file 12933_2023_1782_MOESM1_ESM.docx]

**Supplementary Tables and Figures**

**Table S1**

Clinical characteristics of older and younger subgroups

**Table S2**

Clinical characteristics of female and male subgroups

**Table S3**

Clinical characteristics of patients with complete cases for multiple regression analysis (total)

**Table S4**

Clinical characteristics of older and younger subgroups with complete cases for multiple regression analysis

**Table S5**

Clinical characteristics of female and male subgroups with complete cases for multiple regression analysis

**Figure S1**

Flowchart for the recruitment of the patients

**Figure S2**

Method for measuring myocardial CT values on non-contrasted CT images

**Figure S3**

Multiple logistic regression analyses (total)

**Table S1**

**Clinical characteristics of older and younger subgroups**

|  | Older subgroup | N | Younger subgroup | N | *p* |
| --- | --- | --- | --- | --- | --- |
| Age (years) | 72.8±5.1 | 78 | 56.2±7.6 | 46 | <0.0001 |
| Sex (male/female) | 39/39 | 78 | 33/13 | 46 | 0.0178 |
| BMI (kg/m²) | 25.2±3.8 | 78 | 28.0±4.6 | 46 | 0.0003 |
| Waist circumference (cm) | 92.7±9.5 | 35 | 97.6±10.7 | 26 | 0.0616 |
| Previous highest BMI (kg/m²) | 28.7±4.4 | 54 | 30.5±4.3 | 37 | 0.0480 |
| Systolic blood pressure (mmHg) | 139.4±19.3 | 71 | 145±24 | 40 | 0.2099 |
| Diastolic blood pressure (mmHg) | 74.9±12.9 | 71 | 87±15 | 40 | <0.0001 |
| FPG (mg/dl) | 144±44 | 74 | 154±46 | 46 | 0.2430 |
| HOMA-β | 39.7±43.2 | 44 | 47.1±49.4 | 25 | 0.5186 |
| CPR index | 1.16±0.95 | 49 | 1.46±0.94 | 34 | 0.1704 |
| HOMA-IR | 2.3±1.4 | 44 | 3.3±2.0 | 25 | 0.0241 |
| HbA1c (%; mmol/mol) | 7.9±1.5; 63±16.4 | 78 | 8.4±2.2; 68±24.0 | 46 | 0.1321 |
| Adiponectin (μg/ml) | 9.2±5.5 | 20 | 7.7±5.7 | 16 | 0.4430 |
| T-chol (mg/dl) | 197±32 | 77 | 202±42 | 44 | 0.4690 |
| HDL-chol (mg/dl) | 56±18 | 78 | 53±15 | 46 | 0.4045 |
| LDL-chol (mg/dl) | 117±30 | 74 | 119±35 | 46 | 0.7776 |
| TGs (mg/dl) | 132±63 | 78 | 189±179 | 46 | 0.0104 |
| UA (mg/dl) | 5.3±1.3 | 77 | 5.7±1.3 | 46 | 0.0972 |
| AST (U/L) | 24±16 | 78 | 31±24 | 46 | 0.0571 |
| ALT (U/L) | 23±13 | 78 | 36±26 | 46 | 0.0004 |
| γGTP (U/L) | 42±44 | 77 | 72±97 | 46 | 0.0205 |
| eGFR (ml/min/1.73 m²) | 69.2±17.2 | 74 | 80.1±21.0 | 46 | 0.0025 |
| BNP (pg/ml) | 40.8±45.0 | 47 | 25.1±35.3 | 22 | 0.1562 |
| EF (%) | 68.6±6.4 | 62 | 65.6±5.6 | 30 | 0.0332 |
| IVC_max_ | 11.9±3.2 | 58 | 11.8±4.0 | 28 | 0.9320 |
| Lat e’ (cm/sec) | 7.2±1.5 | 22 | 8.0±1.9 | 11 | 0.2003 |
| Sep E/e’ | 12.5±4.7 | 39 | 11.0±4.0 | 18 | 0.2256 |
| Mean E/e’ | 11.3±2.9 | 25 | 10.4±5.2 | 11 | 0.2152 |
| Wall motion abnormalities (Yes/No) | 5/57 | 62 | 2/28 | 30 | 0.8126 |
| Myocardial CT value (HU) | 47.8±7.5 | 78 | 47.8±5.9 | 46 | 0.9921 |
| Liver CT value (HU) | 55.1±9.4 | 78 | 49.9±12.0 | 46 | 0.0080 |
| Liver CT value minus splenic CT value (HU) | 6.8±9.8 | 78 | 2.6±10.8 | 46 | 0.0281 |
| Pancreatic CT value (HU) | 34.8±9.6 | 76 | 37.5±8.9 | 46 | 0.1247 |
| Pancreatic CT value minus splenic CT value (HU) | -13.6±9.8 | 76 | -9.8±10.5 | 46 | 0.0430 |
| Iliopsoas muscle CT value (HU) | 49.5±5.5 | 77 | 52.2±7.2 | 45 | 0.0234 |
| Splenic CT value (HU) | 48.3±4.0 | 78 | 47.3±5.8 | 46 | 0.2517 |
| VFA (cm²) | 125.7±72.6 | 64 | 141.8±59.5 | 40 | 0.2411 |
| SFA (cm²) | 157.1±81.6 | 58 | 207.3±101.4 | 35 | 0.0104 |
| Coronary arteries with ≥ 50% stenosis in CCTA images (Yes/No) | 52/26 | 78 | 23/23 | 46 | 0.0667 |
| Clinical history of PCI or CABG surgery after the first CCTA (Yes/No) | 27/51 | 78 | 14/32 | 46 | 0.6326 |

Data are presented as the mean ± standard deviation or number of participants (N). BMI, body mass index; HbA1c, the levels of hemoglobin A1c; FPG, fasting plasma glucose; CPI, C-peptide index; HOMA-β, homeostasis model assessment of β-cell function; HOMA-IR, homeostasis model assessment of insulin resistance; T-Chol, total cholesterol; TGs, triglycerides; HDL-C, high-density lipoprotein cholesterol; LDL-C, low-density lipoprotein cholesterol; UA, uric acid; AST, aspartate transaminase; ALT, alanine transaminase; γGTP, γ-glutamyltranspeptidase; eGFR, estimated glomerular filtration rate; BNP, brain natriuretic peptide; EF, ejection fraction; IVC_max_, maximum inferior vena cava diameters; Lat e’, early lateral annular tissue Doppler velocity; Sep E/e’, septum mitral early diastolic velocity/early lateral annular tissue Doppler velocity; Mean E/e’, mean mitral early diastolic velocity/early lateral annular tissue Doppler velocity; VFA, visceral fat area; SFA, subcutaneous fat area; CCTA, coronary computed tomography angiography.

**Table S2**

**Clinical characteristics of female and male subgroups**

|  | Female subgroup | N | Male subgroup | N | *p* |
| --- | --- | --- | --- | --- | --- |
| Age (years) | 69.2±8.9 | 52 | 64.8±10.5 | 72 | 0.0159 |
| BMI (kg/m²) | 25.7±4.6 | 52 | 26.6±4.2 | 72 | 0.2526 |
| Waist circumference (cm) | 93.3±10.2 | 24 | 95.7±10.3 | 37 | 0.3779 |
| Previous highest BMI (kg/m²) | 29.0±4.8 | 40 | 29.7±4.2 | 51 | 0.4646 |
| Systolic blood pressure (mmHg) | 138±18 | 46 | 144±23 | 65 | 0.1752 |
| Diastolic blood pressure (mmHg) | 75±13 | 46 | 82±15 | 65 | 0.0058 |
| FPG (mg/dl) | 145±46 | 50 | 150±44 | 70 | 0.5937 |
| HOMA-β | 46.1±60.8 | 32 | 39.1±26.0 | 37 | 0.5291 |
| CPR index | 1.20±1.01 | 38 | 1.35±0.91 | 45 | 0.4828 |
| HOMA-IR | 2.6±2.1 | 32 | 2.7±1.4 | 37 | 0.9457 |
| HbA1c (%; mmol/mol) | 8.3±1.7; 67±18.6 | 52 | 7.9±1.9; 63±20.8 | 72 | 0.2350 |
| Adiponectin (μg/ml) | 10.6±6.7 | 11 | 7.6±5.0 | 25 | 0.1528 |
| T-chol (mg/dl) | 197±34 | 52 | 199±38 | 69 | 0.7499 |
| HDL-chol (mg/dl) | 58±17 | 52 | 53±17 | 72 | 0.1405 |
| LDL-chol (mg/dl) | 118±34 | 50 | 118±31 | 70 | 0.9901 |
| TGs (mg/dl) | 127±57 | 52 | 172±151 | 72 | 0.0424 |
| UA (mg/dl) | 4.8±1.1 | 52 | 6.0±1.2 | 71 | <0.0001 |
| AST (U/L) | 25±20 | 52 | 28±19 | 72 | 0.4885 |
| ALT (U/L) | 24±19 | 52 | 30±20 | 72 | 0.1140 |
| γGTP (U/L) | 36±41 | 51 | 65±83 | 72 | 0.0255 |
| eGFR (ml/min/1.73 m²) | 73.6±20.3 | 50 | 73.2±18.9 | 70 | 0.9172 |
| BNP (pg/ml) | 35.1±31.1 | 31 | 36.4±50.4 | 38 | 0.9045 |
| EF (%) | 69.1±6.1 | 39 | 66.5±6.1 | 53 | 0.0428 |
| IVC_max_ | 11.0±3.4 | 36 | 11.5±3.4 | 50 | 0.0376 |
| Lat e’ (cm/sec) | 7.3±1.6 | 17 | 7.5±1.8 | 16 | 0.7785 |
| Sep E/e’ | 13.0±5.5 | 23 | 11.4±3.6 | 34 | 0.1987 |
| Mean E/e’ | 10.8±4.1 | 18 | 11.3±3.4 | 18 | 0.1437 |
| Wall motion abnormalities (Yes/No) | 1/38 | 39 | 6/47 | 53 | 0.1175 |
| Myocardial CT value (HU) | 47.1±6.6 | 52 | 48.3±7.1 | 72 | 0.3367 |
| Liver CT value (HU) | 53.8±11.9 | 52 | 52.7±9.8 | 72 | 0.5585 |
| Liver CT value minus splenic CT value (HU) | 5.3±11.7 | 52 | 5.2±9.4 | 72 | 0.9482 |
| Pancreatic CT value (HU) | 36.6±10.1 | 52 | 35.3±8.9 | 70 | 0.4593 |
| Pancreatic CT value minus splenic CT value (HU) | -12.0±9.7 | 52 | -12.4±10.6 | 70 | 0.8433 |
| Iliopsoas muscle CT value (HU) | 50.2±6.4 | 52 | 50.7±6.2 | 70 | 0.6445 |
| Splenic CT value (HU) | 48.5±4.3 | 52 | 47.5±5.0 | 72 | 0.2368 |
| VFA (cm²) | 111.8±54.2 | 47 | 148.5±74.0 | 57 | 0.0056 |
| SFA (cm²) | 189.1±81.3 | 40 | 166.1±99.5 | 53 | 0.2361 |
| Coronary arteries with ≥ 50% stenosis in CCTA images (Yes/No) | 30/22 | 52 | 45/27 | 72 | 0.5889 |
| Clinical history of PCI or CABG surgery after the first CCTA (Yes/No) | 13/39 | 52 | 28/44 | 72 | 0.1048 |

Data are presented as the mean ± standard deviation or number of participants (N). BMI, body mass index; HbA1c, the levels of hemoglobin A1c; FPG, fasting plasma glucose; CPI, C-peptide index; HOMA-β, homeostasis model assessment of β-cell function; HOMA-IR, homeostasis model assessment of insulin resistance; T-Chol, total cholesterol; TGs, triglycerides; HDL-C, high-density lipoprotein cholesterol; LDL-C, low-density lipoprotein cholesterol; UA, uric acid; AST, aspartate transaminase; ALT, alanine transaminase; γGTP, γ-glutamyltranspeptidase; eGFR, estimated glomerular filtration rate; BNP, brain natriuretic peptide; EF, ejection fraction; IVC_max_, maximum inferior vena cava diameters; Lat e’, early lateral annular tissue Doppler velocity; Sep E/e’, septum mitral early diastolic velocity/early lateral annular tissue Doppler velocity; Mean E/e’, mean mitral early diastolic velocity/early lateral annular tissue Doppler velocity; VFA, visceral fat area; SFA, subcutaneous fat area; CCTA, coronary computed tomography angiography.

**Table S3**

**Clinical characteristics of patients with complete cases for multiple regression analysis (total)**

|  |  | N |
| --- | --- | --- |
| Age (years) | 67.4±9.9 | 78 |
| Sex (male/female) | 42/36 | 78 |
| BMI (kg/m²) | 25.5±4.3 | 78 |
| Waist circumference (cm) | 92.4±8.9 | 41 |
| Previous highest BMI (kg/m²) | 28.6±4.5 | 59 |
| Systolic blood pressure (mmHg) | 144±21 | 71 |
| Diastolic blood pressure (mmHg) | 79±15 | 71 |
| FPG (mg/dl) | 149±46 | 74 |
| HOMA-β | 43.0±51.1 | 46 |
| CPR index | 1.35±1.07 | 57 |
| HOMA-IR | 2.7±1.9 | 46 |
| HbA1c (%; mmol/mol) | 8.1±1.6; 65±17.5 | 78 |
| Adiponectin (μg/ml) | 8.6±5.6 | 27 |
| T-chol (mg/dl) | 200±39 | 78 |
| HDL-chol (mg/dl) | 56±19 | 78 |
| LDL-chol (mg/dl) | 118±35 | 77 |
| TGs (mg/dl) | 154±120 | 78 |
| UA (mg/dl) | 5.4±1.3 | 77 |
| AST (U/L) | 24±14 | 78 |
| ALT (U/L) | 25±16 | 78 |
| γGTP (U/L) | 43±44 | 78 |
| eGFR (ml/min/1.73 m²) | 74.7±20.3 | 75 |
| BNP (pg/ml) | 41.9±47.8 | 47 |
| EF (%) | 68.2±5.8 | 78 |
| IVC_max_ | 11.9±3.7 | 73 |
| Lat e’ (cm/sec) | 7.4±1.7 | 32 |
| Sep E/e’ | 12.2±4.7 | 44 |
| Mean E/e’ | 11.0±3.7 | 36 |
| Wall motion abnormalities (Yes/No) | 5/73 | 78 |
| Myocardial CT value (HU) | 47.4±6.3 | 78 |
| Liver CT value (HU) | 54.9±9.2 | 78 |
| Liver CT value minus splenic CT value (HU) | 6.4±9.3 | 78 |
| Pancreatic CT value (HU) | 36.7±9.2 | 76 |
| Pancreatic CT value minus splenic CT value (HU) | -11.9±9.6 | 76 |
| Iliopsoas muscle CT value (HU) | 50.5±65.9 | 78 |
| Splenic CT value (HU) | 48.5±4.7 | 78 |
| VFA (cm²) | 127.1±70.8 | 78 |
| SFA (cm²) | 167.6±80.6 | 73 |
| Coronary arteries with ≥ 50% stenosis in CCTA images (Yes/No) | 54/24 | 78 |
| Clinical history of PCI or CABG surgery after the first CCTA (Yes/No) | 33/45 | 78 |

Data are presented as the mean ± standard deviation or number of participants (N). BMI, body mass index; HbA1c, the levels of hemoglobin A1c; FPG, fasting plasma glucose; CPI, C-peptide index; HOMA-β, homeostasis model assessment of β-cell function; HOMA-IR, homeostasis model assessment of insulin resistance; T-Chol, total cholesterol; TGs, triglycerides; HDL-C, high-density lipoprotein cholesterol; LDL-C, low-density lipoprotein cholesterol; UA, uric acid; AST, aspartate transaminase; ALT, alanine transaminase; γGTP, γ-glutamyltranspeptidase; eGFR, estimated glomerular filtration rate; BNP, brain natriuretic peptide; EF, ejection fraction; IVC_max_, maximum inferior vena cava diameters; Lat e’, early lateral annular tissue Doppler velocity; Sep E/e’, septum mitral early diastolic velocity/early lateral annular tissue Doppler velocity; Mean E/e’, mean mitral early diastolic velocity/early lateral annular tissue Doppler velocity; VFA, visceral fat area; SFA, subcutaneous fat area; CCTA, coronary computed tomography angiography.

**Table S4**

**Clinical characteristics of older and younger subgroups with complete cases for multiple regression analysis**

Clinical characteristics of older and younger subgroups with complete cases for multiple regression analysis for ejection fraction (EF)

|  | Older subgroup | N | Younger subgroup | N | *p* |
| --- | --- | --- | --- | --- | --- |
| Age (years) | 72.7±5.3 | 52 | 56.7±8.1 | 26 | <0.0001 |
| Sex (male/female) | 24/28 | 52 | 18/8 | 26 | 0.0539 |
| BMI (kg/m²) | 24.6±3.9 | 52 | 27.4±4.6 | 26 | 0.0075 |
| Waist circumference (cm) | 91.6±9.5 | 28 | 94.2±7.4 | 13 | 0.3848 |
| Previous highest BMI (kg/m²) | 27.9±4.3 | 39 | 30.1±4.5 | 20 | 0.0666 |
| Systolic blood pressure (mmHg) | 140.2±20.1 | 48 | 151±22 | 23 | 0.0382 |
| Diastolic blood pressure (mmHg) | 74.5±13.0 | 48 | 89±15 | 23 | 0.0001 |
| FPG (mg/dl) | 143±43 | 48 | 160±52 | 26 | 0.1485 |
| HOMA-β | 42.6±48.1 | 33 | 43.8±59.9 | 13 | 0.9442 |
| CPR index | 1.25±1.06 | 36 | 1.52±1.09 | 21 | 0.3638 |
| HOMA-IR | 2.3±1.5 | 33 | 3.9±2.4 | 13 | 0.0079 |
| HbA1c (%; mmol/mol) | 7.9±1.5; 63±16.4 | 52 | 8.4±1.8; 68±19.7 | 26 | 0.1710 |
| Adiponectin (μg/ml) | 9.1±5.1 | 17 | 7.7±6.5 | 10 | 0.5214 |
| T-chol (mg/dl) | 198±35 | 52 | 206±47 | 26 | 0.4139 |
| HDL-chol (mg/dl) | 58±20 | 52 | 53±16 | 26 | 0.3530 |
| LDL-chol (mg/dl) | 117±33 | 31 | 122±39 | 26 | 0.5222 |
| TGs (mg/dl) | 131±63 | 52 | 199±181 | 26 | 0.0177 |
| UA (mg/dl) | 5.2±1.3 | 51 | 5.7±1.3 | 26 | 0.1008 |
| AST (U/L) | 22±7 | 52 | 27±22 | 26 | 0.1906 |
| ALT (U/L) | 22±11 | 78 | 30±23 | 26 | 0.0297 |
| γGTP (U/L) | 35±27 | 52 | 60±63 | 26 | 0.0157 |
| eGFR (ml/min/1.73 m²) | 71.0±16.8 | 49 | 81.7±24.7 | 26 | 0.0298 |
| BNP (pg/ml) | 46.8±50.5 | 32 | 31.7±41.4 | 15 | 0.3193 |
| EF (%) | 69.0±6.0 | 52 | 66.5±5.2 | 26 | 0.0781 |
| IVC_max_ | 12.1±3.4 | 49 | 11.6±4.2 | 24 | 0.6602 |
| Lat e’ (cm/sec) | 7.2±1.5 | 22 | 8.0±2.0 | 10 | 0.2191 |
| Sep E/e’ | 12.6±4.9 | 29 | 11.2±4.2 | 15 | 0.3453 |
| Mean E/e’ | 11.3±2.9 | 25 | 10.4±5.2 | 11 | 0.5220 |
| Wall motion abnormalities (Yes/No) | 3/49 | 52 | 2/24 | 26 | 0.7478 |
| Myocardial CT value (HU) | 47.1±6.8 | 52 | 48.1±5.3 | 26 | 0.5106 |
| Liver CT value (HU) | 56.0±9.1 | 52 | 52.7±9.2 | 26 | 0.1406 |
| Liver CT value minus splenic CT value (HU) | 7.7±9.3 | 52 | 3.6±8.8 | 26 | 0.0658 |
| Pancreatic CT value (HU) | 35.8±8.8 | 50 | 38.6±9.8 | 26 | 0.2093 |
| Pancreatic CT value minus splenic CT value (HU) | -12.7±8.7 | 50 | -10.5±11.1 | 26 | 0.3532 |
| Iliopsoas muscle CT value (HU) | 49.5±4.9 | 52 | 52.4±7.3 | 26 | 0.0403 |
| Splenic CT value (HU) | 48.2±3.7 | 52 | 49.1±6.2 | 26 | 0.4639 |
| VFA (cm²) | 126.5±78.2 | 52 | 128.5±54.7 | 26 | 0.9068 |
| SFA (cm²) | 156.9±82.4 | 49 | 189.4±73.7 | 24 | 0.1061 |
| Coronary arteries with ≥ 50% stenosis in CCTA images (Yes/No) | 38/14 | 52 | 16/10 | 26 | 0.2980 |
| Clinical history of PCI or CABG surgery after the first CCTA (Yes/No) | 23/29 | 52 | 10/16 | 26 | 0.6268 |

Clinical characteristics of the older subgroup with complete cases for multiple regression analysis early lateral annular tissue Doppler velocity (Lat e')

|  | Older subgroup | N |
| --- | --- | --- |
| Age (years) | 72.7±5.1 | 22 |
| Sex (male/female) | 8/14 | 22 |
| BMI (kg/m²) | 22.9±3.1 | 22 |
| Waist circumference (cm) | 88.4±9.4 | 9 |
| Previous highest BMI (kg/m²) | 26.8±2.93 | 17 |
| Systolic blood pressure (mmHg) | 135.0±20.0 | 21 |
| Diastolic blood pressure (mmHg) | 70.5±11.5 | 21 |
| FPG (mg/dl) | 134±36 | 20 |
| HOMA-β | 28.6±20.3 | 13 |
| CPR index | 0.91±0.43 | 13 |
| HOMA-IR | 1.4±0.7 | 13 |
| HbA1c (%; mmol/mol) | 8.2±1.9; 66±20.8 | 22 |
| T-chol (mg/dl) | 192±33 | 22 |
| HDL-chol (mg/dl) | 57±23 | 22 |
| LDL-chol (mg/dl) | 108±34 | 21 |
| TGs (mg/dl) | 139±77 | 22 |
| UA (mg/dl) | 5.0±1.5 | 22 |
| AST (U/L) | 20±6 | 22 |
| ALT (U/L) | 18±9 | 22 |
| γGTP (U/L) | 31±23 | 22 |
| eGFR (ml/min/1.73 m²) | 75.8±16.4 | 22 |
| BNP (pg/ml) | 53.6±60.3 | 20 |
| EF (%) | 70.2±6.8 | 22 |
| IVC_max_ | 12.7±3.9 | 21 |
| Lat e’ (cm/sec) | 7.2±1.5 | 22 |
| Sep E/e’ | 13.4±2.3 | 12 |
| Mean E/e’ | 11.3±2.9 | 22 |
| Wall motion abnormalities (Yes/No) | 1/21 | 22 |
| Myocardial CT value (HU) | 50.3±4.3 | 22 |
| Liver CT value (HU) | 58.0±7.0 | 22 |
| Liver CT value minus splenic CT value (HU) | 9.8±6.6 | 22 |
| Pancreatic CT value (HU) | 38.1±7.0 | 22 |
| Pancreatic CT value minus splenic CT value (HU) | -10.2±7.3 | 21 |
| Iliopsoas muscle CT value (HU) | 49.0±4.5 | 22 |
| Splenic CT value (HU) | 48.2±4.2 | 22 |
| VFA (cm²) | 95.9±41.3 | 22 |
| SFA (cm²) | 124.4±61.5 | 19 |
| Coronary arteries with ≥ 50% stenosis in CCTA images (Yes/No) | 18/4 | 22 |
| Clinical history of PCI or CABG surgery after the first CCTA (Yes/No) | 12/10 | 22 |

Data are presented as the mean ± standard deviation or number of participants (N). BMI, body mass index; HbA1c, the levels of hemoglobin A1c; FPG, fasting plasma glucose; CPI, C-peptide index; HOMA-β, homeostasis model assessment of β-cell function; HOMA-IR, homeostasis model assessment of insulin resistance; T-Chol, total cholesterol; TGs, triglycerides; HDL-C, high-density lipoprotein cholesterol; LDL-C, low-density lipoprotein cholesterol; UA, uric acid; AST, aspartate transaminase; ALT, alanine transaminase; γGTP, γ-glutamyltranspeptidase; eGFR, estimated glomerular filtration rate; BNP, brain natriuretic peptide; EF, ejection fraction; IVC_max_, maximum inferior vena cava diameters; Lat e’, early lateral annular tissue Doppler velocity; Sep E/e’, septum mitral early diastolic velocity/early lateral annular tissue Doppler velocity; Mean E/e’, mean mitral early diastolic velocity/early lateral annular tissue Doppler velocity; VFA, visceral fat area; SFA, subcutaneous fat area; CCTA, coronary computed tomography angiography.

**Table S5**

**Clinical characteristics of female and male subgroups with complete cases for multiple regression analysis**

Clinical characteristics of female and male subgroups with complete cases for multiple regression analysis for ejection fraction (EF)

|  | Female subgroup | N | Male subgroup | N | *p* |
| --- | --- | --- | --- | --- | --- |
| Age (years) | 68.9±9.3 | 36 | 66.1±10.4 | 42 | 0.2215 |
| BMI (kg/m²) | 24.9±4.4 | 36 | 26.1±4.2 | 42 | 0.2217 |
| Waist circumference (cm) | 90.1±7.4 | 16 | 94.0±9.6 | 25 | 0.1772 |
| Previous highest BMI (kg/m²) | 28.1±4.8 | 27 | 29.1±4.1 | 32 | 0.4260 |
| Systolic blood pressure (mmHg) | 138±20 | 30 | 148±22 | 41 | 0.0373 |
| Diastolic blood pressure (mmHg) | 74±14 | 30 | 83±15 | 41 | 0.0130 |
| FPG (mg/dl) | 143±45 | 34 | 154±48 | 40 | 0.3171 |
| HOMA-β | 52.1±70.0 | 23 | 33.8±16.0 | 23 | 0.2268 |
| CPR index | 1.31±1.13 | 26 | 1.38±1.03 | 31 | 0.7835 |
| HOMA-IR | 2.9±2.3 | 23 | 2.6±1.4 | 23 | 0.7159 |
| HbA1c (%; mmol/mol) | 8.3±1.8; 67±19.7 | 52 | 7.9±1.5; 63±16.4 | 42 | 0.3118 |
| Adiponectin (μg/ml) | 9.6±6.2 | 10 | 8.0±5.3 | 17 | 0.4728 |
| T-chol (mg/dl) | 201±35 | 36 | 200±43 | 42 | 0.8952 |
| HDL-chol (mg/dl) | 58±19 | 36 | 54±20 | 42 | 0.3772 |
| LDL-chol (mg/dl) | 119±37 | 35 | 118±33 | 42 | 0.8241 |
| TGs (mg/dl) | 134±61 | 36 | 171±152 | 42 | 0.1671 |
| UA (mg/dl) | 4.7±1.1 | 36 | 6.0±1.1 | 41 | <0.0001 |
| AST (U/L) | 20±5 | 36 | 27±18 | 42 | 0.0309 |
| ALT (U/L) | 20±10 | 36 | 29±19 | 42 | 0.0085 |
| γGTP (U/L) | 25±11 | 36 | 59±54 | 42 | 0.0004 |
| eGFR (ml/min/1.73 m²) | 75.3±20.4 | 34 | 74.1±20.5 | 41 | 0.7905 |
| BNP (pg/ml) | 43.4±33.7 | 21 | 40.8±57.4 | 26 | 0.8513 |
| EF (%) | 69.7±5.4 | 36 | 66.9±5.9 | 42 | 0.0342 |
| IVC_max_ | 11.0±3.4 | 24 | 12.8±3.7 | 39 | 0.0381 |
| Lat e’ (cm/sec) | 7.3±1.6 | 17 | 7.5±1.8 | 15 | 0.8182 |
| Sep E/e’ | 12.7±5.5 | 20 | 11.7±4.0 | 24 | 0.5050 |
| Mean E/e’ | 11.3±3.3 | 20 | 10.7±34.0 | 19 | 0.6722 |
| Wall motion abnormalities (Yes/No) | 0/36 | 36 | 5/37 | 42 | 0.0324 |
| Myocardial CT value (HU) | 46.6±6.5 | 36 | 48.1±6.2 | 42 | 0.2831 |
| Liver CT value (HU) | 56.1±9.6 | 36 | 53.8±8.9 | 42 | 0.2763 |
| Liver CT value minus splenic CT value (HU) | 7.5±10.2 | 36 | 5.4±8.4 | 42 | 0.3320 |
| Pancreatic CT value (HU) | 36.5±10.5 | 36 | 36.9±7.9 | 40 | 0.8530 |
| Pancreatic CT value minus splenic CT value (HU) | -12.1±10.0 | 36 | -11.7±9.3 | 40 | 0.8618 |
| Iliopsoas muscle CT value (HU) | 49.5±6.2 | 36 | 51.3±5.6 | 42 | 0.1897 |
| Splenic CT value (HU) | 48.6±4.3 | 36 | 48.4±5.0 | 42 | 0.8282 |
| VFA (cm²) | 107.3±47.4 | 36 | 144.2±82.8 | 42 | 0.0209 |
| SFA (cm²) | 185.3±76.6 | 33 | 153.1±81.8 | 40 | 0.0893 |
| Coronary arteries with ≥ 50% stenosis in CCTA images (Yes/No) | 22/14 | 36 | 32/10 | 42 | 0.1503 |
| Clinical history of PCI or CABG surgery after the first CCTA (Yes/No) | 13/23 | 36 | 20/22 | 42 | 0.3051 |

Clinical characteristics of the female subgroup with complete cases for multiple regression analysis early lateral annular tissue Doppler velocity (Lat e')

|  | Female subgroup | N |
| --- | --- | --- |
| Age (years) | 69.4±8.9 | 17 |
| BMI (kg/m²) | 23.7±5.1 | 17 |
| Waist circumference (cm) | 86.1±7.9 | 7 |
| Previous highest BMI (kg/m²) | 26.9±4.7 | 13 |
| Systolic blood pressure (mmHg) | 134.0±23.0 | 15 |
| Diastolic blood pressure (mmHg) | 70.6±14.9 | 15 |
| FPG (mg/dl) | 132±30 | 16 |
| HOMA-β | 49.4±65.6 | 11 |
| CPR index | 1.44±1.45 | 12 |
| HOMA-IR | 2.1±1.8 | 11 |
| HbA1c (%; mmol/mol) | 8.2±1.9; 66±20.8 | 17 |
| T-chol (mg/dl) | 205±35 | 17 |
| HDL-chol (mg/dl) | 60±23 | 17 |
| LDL-chol (mg/dl) | 119±41 | 16 |
| TGs (mg/dl) | 141±72 | 17 |
| UA (mg/dl) | 4.4±1.2 | 17 |
| AST (U/L) | 19±6 | 17 |
| ALT (U/L) | 19±12 | 17 |
| γGTP (U/L) | 24±11 | 17 |
| eGFR (ml/min/1.73 m²) | 75.5±18.0 | 17 |
| BNP (pg/ml) | 43.0±38.9 | 13 |
| EF (%) | 71.4±5.2 | 17 |
| IVC_max_ | 10.6±3.7 | 17 |
| Lat e’ (cm/sec) | 7.3±1.6 | 17 |
| Sep E/e’ | 12.7±2.2 | 8 |
| Mean E/e’ | 11.4±3.5 | 17 |
| Wall motion abnormalities (Yes/No) | 0/17 | 17 |
| Myocardial CT value (HU) | 50.0±4.4 | 17 |
| Liver CT value (HU) | 57.6±7.0 | 17 |
| Liver CT value minus splenic CT value (HU) | 8.1±8.4 | 17 |
| Pancreatic CT value (HU) | 40.6±5.9 | 17 |
| Pancreatic CT value minus splenic CT value (HU) | -8.9±4.8 | 17 |
| Iliopsoas muscle CT value (HU) | 50.2±7.5 | 17 |
| Splenic CT value (HU) | 49.5±5.6 | 17 |
| VFA (cm²) | 91.2±35.3 | 17 |
| SFA (cm²) | 152.7±59.8 | 14 |
| Coronary arteries with ≥ 50% stenosis in CCTA images (Yes/No) | 12/5 | 17 |
| Clinical history of PCI or CABG surgery after the first CCTA (Yes/No) | 6/11 | 17 |

Data are presented as the mean ± standard deviation or number of participants (N). BMI, body mass index; HbA1c, the levels of hemoglobin A1c; FPG, fasting plasma glucose; CPI, C-peptide index; HOMA-β, homeostasis model assessment of β-cell function; HOMA-IR, homeostasis model assessment of insulin resistance; T-Chol, total cholesterol; TGs, triglycerides; HDL-C, high-density lipoprotein cholesterol; LDL-C, low-density lipoprotein cholesterol; UA, uric acid; AST, aspartate transaminase; ALT, alanine transaminase; γGTP, γ-glutamyltranspeptidase; eGFR, estimated glomerular filtration rate; BNP, brain natriuretic peptide; EF, ejection fraction; IVC_max_, maximum inferior vena cava diameters; Lat e’, early lateral annular tissue Doppler velocity; Sep E/e’, septum mitral early diastolic velocity/early lateral annular tissue Doppler velocity, Mean E/e’, mean mitral early diastolic velocity/early lateral annular tissue Doppler velocity; VFA, visceral fat area; SFA, subcutaneous fat area; CCTA, coronary computed tomography angiography.

**Figure S1**

Patients with type 2 diabetes who were referred for ECG-gated coronary computed tomography angiography (CCTA) examinations for the first time and also underwent abdominal computed tomography (CT) scans within 1 year of CCTA between January 2000 and March 2021 (N= 411)

Heart failure with reduced ejection fraction (≤ 40%) (N=31)

Valvular heart disease (N=13)

Previous history of percutaneous coronary intervention (PCI) for coronary artery disease (N=78)

Liver cirrhosis (N=39)

Renal failure (N=2)

Malignant diseases (N=96)

Glucocorticoids use (N=22)

CCTA or abdominal CT examinations which were not performed at 120 kV (N=6)

N=124

Excluded (N=287)

**Flowchart for the recruitment of the patients**

**Figure S2**

**Method for measuring myocardial CT values on non-contrasted CT images**

**
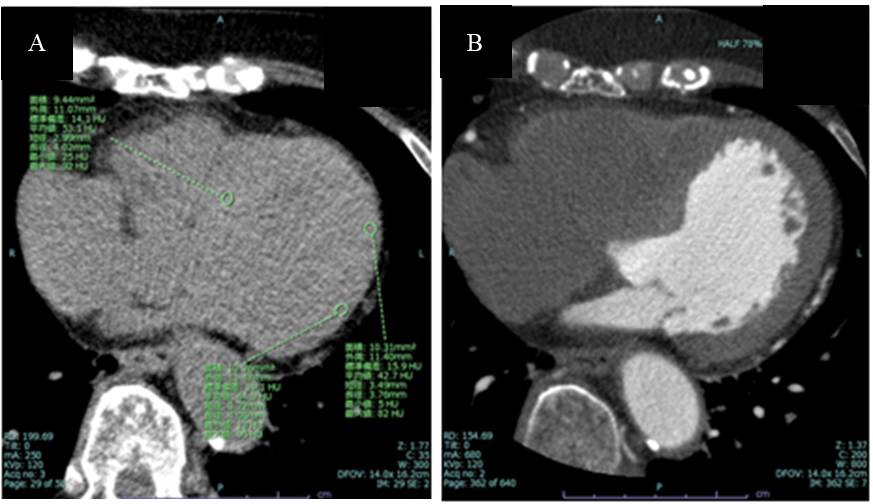
**

Myocardial CT value was defined as the mean CT value of three regions of interest with areas of 10 mm^2^ in two different parts of the left ventricular free wall and one part of the myocardial septum. To confirm the intramyocardial measurement areas excluding blood pool and epicardial fat, we compared noncontrasted (A) and contrasted (B) images side-by-side. The green circles illustrate how the CT value measurements were performed.

**Figure S3**

**Multiple logistic regression analyses (total)**





**A B**

60

60

Myocardial CT value (HU)

50

Myocardial CT value (HU)

50

40

40

30

30

*p*=0.6041

*p*=0.4175

No

No

Yes

Yes

Coronary arteries with ≥ 50% stenosis in CCTA images

Coronary arteries with ≥ 50% stenosis in CCTA images

The multiple logistic regression analyses of the relationship between myocardial CT value (an index of myocardial fat content) and coronary arteries with ≥ 50% stenosis in CCTA images (A) and clinical history of PCI or CABG surgery after the first CCTA examinations (B). There was no significant association between myocardial CT value and coronary arteries with ≥ 50% stenosis in CCTA images. Likewise, there was no significant association between H and clinical history of PCI or CABG surgery after the first CCTA examinations. CCTA, coronary computed tomography angiography; PCI, percutaneous coronary intervention; CABG, coronary artery bypass graft.
